# Sleep Patterns and Quality among Young Adults of Karachi, Pakistan

**DOI:** 10.5334/jcr.260

**Published:** 2026-01-14

**Authors:** Muhammad Taha Abid, Mudassir Abbas, Gati Ara, Nimrah Iqbal, Turba Naz, Areeka Irfan

**Affiliations:** 1 MBBS Student, Dow Medical College, Dow University of Health Sciences, PK; 2 Assistant Professor, Department of Community Medicine, Dow University of Health Sciences, PK

**Keywords:** sleep, sleep patterns, quality of sleep, PSQI, ESS, awakening-sleep rhythm

## Abstract

**Introduction:**

Sleep plays a vital role in maintaining overall health, supporting processes like restoration, memory consolidation, emotional regulation, and metabolism. However, many people remain unaware of their sleep needs, leading to poor sleep quality and patterns that can negatively impact health, workplace performance, and daily life. Sleep behaviors are shaped by various factors, including cultural practices, urbanization, and lifestyle changes. This study focuses on adults in Karachi, Pakistan, where religious routines and modern living create unique sleep challenges. By exploring these patterns, we aim to identify ways to enhance sleep quality and promote better well-being in this population.

**Methodology:**

This cross-sectional study was conducted in Karachi, Pakistan, over one year, with 400 adults aged 18–64 were recruited. Exclusion criteria included conditions affecting sleep, chronic illnesses, and use of sleep aids. Data collection employed a Google Forms-based structured questionnaire covering demographics, religious practices, and sleep habits. Sleep quality and daytime sleepiness were assessed using the Pittsburgh Sleep Quality Index (PSQI) and Epworth Sleepiness Scale (ESS), respectively. Data analysis, performed in SPSS, included descriptive statistics and hypothesis testing using Chi-squared, Mann–Whitney, and Kruskal–Wallis tests. Sleep patterns were categorized as monophasic, biphasic, or polyphasic using conditional formulas in Excel.

**Results:**

The mean age of participants (n = 384) was 29 ± 0.1 years, with 66.4% women. The most common sleep pattern was monophasic (40.4%), followed by biphasic-siesta (21.6%), biphasic-dawn (20.8%), and polyphasic (17.2%). Women favored segmented sleep patterns (p = 0.019). Biphasic-dawn sleepers reported the least daytime sleepiness (ESS = 7.18 ± 3.85, p = 0.024), while biphasic-siesta sleepers had the poorest sleep quality (PSQI = 6.94 ± 3.01, p = 0.013). Men had better sleep quality (PSQI = 5.22 ± 2.70) and lower daytime sleepiness (ESS = 6.89 ± 3.65) than women (p < 0.001). Weekend sleep disruptions were significant (p < 0.001).

**Conclusions:**

Monophasic sleep dominates, though gender differences suggest women prefer segmented patterns. Biphasic-dawn sleep aligns with better quality and less daytime sleepiness, while biphasic-siesta sleepers experience the poorest outcomes. Men and employed individuals generally report better sleep metrics. Morning prayers improve sleep quality slightly. Students exhibit higher daytime sleepiness and worse sleep quality compared to others.

## Introduction

Sleep is a fundamental constituent of our circadian rhythm, marked by various autonomic nervous activities. This physiological process is unique to everyone – occupying about one-third of their lives. Sleep provides the restoration and repair of major body systems, including cardiovascular, respiratory, musculoskeletal, and central nervous systems [1]. It is a reversible behavioral state of perceptual disengagement from and to the environment [2].

Sleep plays a crucial role in emotion and metabolic regulation, memory consolidation, performance, learning and brain recovery processes. Despite compelling evidence linking insufficient sleep to countless health issues, many remain unaware of the required amount of sleep to prevent deprivation and the adverse outcomes of this on their health. According to the 2006 Institute of Medicine (IOM) report, adults need 7 to 8 hours of sleep per night, while adolescents require a minimum of 9 hours.

When it comes to sleep, both quality and quantity are important. Sleep quality is a measure of an individual’s overall satisfaction with all aspects of their sleep experience. A systematic review and meta-analysis revealed that better sleep quality is positively associated with various health outcomes, including cardiovascular disease, metabolic syndrome, mental health, and dementia [4]. There are four main attributes of sleep quality: sleep efficiency, sleep latency, sleep duration, and wakefulness after sleep onset. High-quality sleep results in feeling rested, having normal reflexes, and maintaining positive relationships. Conversely, poor sleep quality leads to fatigue, irritability, slowed reactions, increased caffeine or alcohol intake, and day-time sleepiness [5].

Daytime sleepiness has emerged as one of the major consequences of insufficient sleep, which has resulted in decreased alertness and slow reaction time. This can lead to occupational and medical errors, workplace injuries, impaired driving, and motor vehicle accidents [3].

Another important variable of healthy sleep is the sleep pattern – directed by the body’s natural circadian rhythm. A healthy sleep pattern depends on personal satisfaction, sleep timing, duration, efficiency, and daytime alertness [6]. Sleep patterns are commonly divided into three categories – monophasic, biphasic, and polyphasic.

The monophasic pattern refers to sleeping only once a day, typically during night, and is the most common sleeping pattern, especially in the most developed societies [7]. The biphasic patterns include two sleeping sessions per day, mostly compromising of a longer sleeping duration at night (6–7 hours) and a short nap during the day (up to 1 hour). This pattern is prevalent in Spain and many Latin American countries [8]. The third type, polyphasic sleep, contains multiple sleep periods throughout the day, including a short night sleep of no more than 3 hours and 2–3 daytime naps of 20–30 minutes each. This pattern is typically prevalent in working population and students to reduce sleep duration to increase working hours [9].

The sleeping patterns can be influenced by several cultural and socioeconomic factors. Studies show crucial evidence that religious observance involves social rules and beliefs that can impact sleep behavior, quality, and outcomes [10]. Traditional prayer times affect sleep duration for observant Muslims, who often wake up early for early morning prayers (such as Fajar and Tahajud) but cannot always go to bed early enough to get adequate sleep. Religion also prescribes preferred sleep positions, which participants found advantageous. A study revealed that religious individuals’ deep faith and practice of praying helped them reduce stress and enhance sleep quality [11]. However, among Muslims, missing prayers could lead to feelings of guilt and unease, negatively influencing sleep quality. Religion affects the sleep of people following Islamic traditions, with these effects varying based on individual religious practices.

Furthermore, recent lifestyle modifications due to rapid economic development and frequent use of modern technology by the local population, integrated with their religious obligations have resulted in sleeping habits that may differ from those of other populations worldwide [12]. Studies have revealed that urban populations experience more adverse sleep effects, including increased daytime sleepiness, inadequate overall sleep quality, increased levels of fatigue, and heightened anxiety compared to rural populations [13].

This research is aimed to assess the sleeping patterns and quality among adults of Karachi, Pakistan. And the influence of religious and cultural beliefs on their overall sleep health. By understanding such factors, interventions can be developed to promote better sleep quality and overall well-being in these populations.

## Methods

### Study Design and population

This cross-sectional study was conducted in Karachi, Pakistan for a period of one year from June 2023 to May 2024. The sample comprised 400 adults aged 18–64 years, selected through convenience sampling. Participants were recruited from various neighborhoods, ensuring a mix of residential types, including apartments and houses, and varying levels of urban density. Inclusion criteria included Muslim adults of both genders residing in Karachi aged between 18 and 64 years and conscious to respond to questions. Pregnant or lactating women, individuals regularly using sleep aids, pain relievers, or anaesthetics, those who had undergone surgery within last six months, individuals undergoing treatment for chronic illnesses such as chemotherapy or radiotherapy, people with diagnosed arthritis, fibromyalgia or other painful conditions, and participants already diagnosed with temporary conditions disrupting normal sleep patterns, such as insomnia, sleep apnoea, or depression were excluded.

### Data collection

Data collection was carried out through an interview-based approach using Google Forms-based questionnaires to record data. The semi-structured questionnaire covered socio-demographic information, religious practices, sleep habits, and associated factors. The questionnaire included questions on sleep duration, sleep quality, frequency and timing of prayers, and perceived sleep disturbances. Participants’ adherence to Islamic prayer times was recorded, with specific attention to Fajr and Tahajjud prayers. Lastly, separate sections were created to assess their daytime sleepiness and overall sleep quality using Epworth Sleepiness Scale (ESS) and Pittsburgh Sleep Quality Index (PSQI) scores through standardized questions.

Epworth Sleepiness Scale (ESS): it is a unidimensional scale with eight items investigating the same domain of how likely a person is to fall asleep or doze off in certain situations. Each item is a four-point Likert scale ranging from zero (would never doze off) to three (high chance of dozing).

Pittsburgh Sleep Quality Index (PSQI): it is a validated tool that relates to the subject’s usual sleep habits during the past month. It consists of nine questions, with subcategories – where questions one to four being open ended, and five to nine based on score, with ranging values from zero to three.

Both scales have validated Urdu translations available on request. Permission to use both these scales was obtained from the developers, due to potential copyright infringement.

### Sample Size

The sample size calculation was performed using OpenEpi, based on a population size of one million and an assumed frequency of the outcome in the population set at 50% due to a lack of prior data. The initial minimum sample size required was determined to be 384, with a confidence level of 95% and a margin of error of 5%. After accounting for a non-response rate of 4%, the final adjusted sample size for the study was set at 400 participants. A total 384 valid responses were gathered.

All participants provided informed written consent, and the study ensured confidentiality and voluntary participation.

### Data Analysis

Data entry and analysis were performed using SPSS version 24. Descriptive statistics, such as mean and standard deviation, were used for quantitative variables like age and scores. Percentages and frequencies were utilized for qualitative variables like gender, educational status, working status, and profession. Participants were compared for any differences associated with respect to sleep patterns and quality. Prevalence and frequencies were expressed as percentages.

The relationship between the two categorical variables was established using Chi-squared tests, while the comparison of mean scores (e.g., ESS and PSQI) against categorical variables (e.g., gender and occupations) were compared using Mann–Whitney tests. Kruskal–Wallis one-way analysis of variance was used to compare the duration of sleep and mean ESS and PSQI scores between the sleep patterns. A p-value (two-tailed) of less than 0.05 was considered as statistically significant.

### Sleep Pattern’s Measurement

We recorded the data obtained from the questionnaire into MS Excel, with each participant’s response occupying a set row. Columns were assigned for variables including sleep and waking times (both weekdays and weekends), nap habits, prayer routines, and qualitative responses e.g., satisfaction and perceived sleep quality. To further streamline the analysis, time-based data was converted into second or decimal hours. Furthermore, Boolean and conditional formulas (“IF”, “AND”, “OR”) were employed to automate value assignments based on specific conditions.

Sleep quality was assessed using a categorical scale based on participant’s responses about the total sleep duration and subjective sleep evaluations. A final score was calculated using conditional formulas.

To classify participants’ sleep patterns, a series of conditional formulas (IF, AND, OR) were applied based on variable related to napping habits and prayer schedules. Sleep patterns were initially classified into monophasic, biphasic, and polyphasic categories using conditions that combined responses on napping and night prayer practices.

### Ethical Considerations

The study received ethical approval from the Institutional Review Board (IRB) of Dow University of Health Sciences (Ref No: IRB-3002/DUHS/Approval/2023/312). It was conducted in accordance with the principles of the Declaration of Helsinki for research involving human participants. Informed consent was obtained through a debriefing statement embedded in the Google Forms, ensuring transparency and clear communication. Participants were informed about the objectives of the study, the voluntary nature of their participation, and the measures taken to safeguard their privacy and confidentiality. They were also reminded of their right to withdraw from the study at any stage without penalty. To protect participant confidentiality, all data were pseudonymized prior to entry and securely stored on password-protected computers and servers, accessible only to the research investigators. Personal identifiers, including names and contact details, were not linked to the dataset, and published findings contain no information that could identify individual participants. The results of the study were subsequently shared with participants via email. Individuals with poor sleep were given advice on improving their sleep and referred for professional guidance.

## Results

### Demographic Description

Mean age of n = 384 participants was 29 +/–0.1 years. Among the people, two-thirds were women (66.4%) while one-third were male (33.6%). Furthermore, students were the most common occupational pool in our study with more than two-thirds (68.0%) making up the study sample, followed by employed individuals (24.7%) and then unemployed (7.3%). Furthermore, the most common age group in our study were that of young adults e.g., 18–34 years (84.7%). No age-related gender bias was observed in the study (p = 0.133). More information can be found on Table 1.

**Table 1:** Distribution of Pakistani Adults According to Their Socio-Demographic Characteristics.


CHARACTERISTICS	MALE (N = 129)	FEMALE (N = 255)

	NO. (%)	NO. (%)

**Age range**		

18–34	111 (86)	214 (83.9)

35–49	10 (7.8)	33 (12.9)

50–64	8 (6.2)	8 (3.1)

**Employment**		

Student	77 (59.7)	184 (72.2)

Employed	50 (38.8)	45 (17.6)

Unemployed	2 (1.6)	26 (10.2)


### Sleep Characteristics

**Figure 1:**
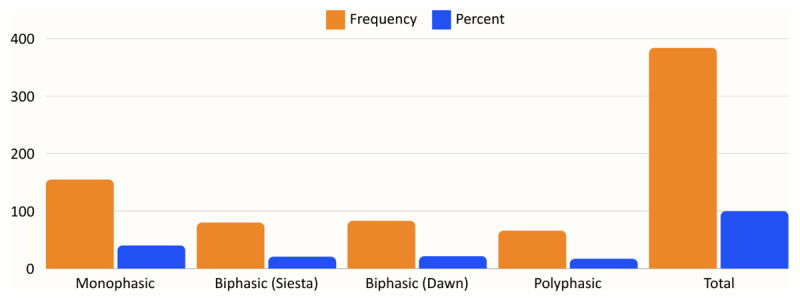
Sleep Pattern Distribution in the Sample Population.

The most common sleep pattern in our study was revealed to be monophasic, with more than two-thirds of the people having this type of sleep (40.4%) followed by biphasic-siesta (21.6%), biphasic-dawn (20.8%), and finally polyphasic (17.2%) sleep patterns. This finding was true across people of all age groups and professions (see Figure 1).

However, gender-based analysis showed women were more likely to have segmented sleep patterns – where the most common sleep pattern in women was biphasic-siesta followed by polyphasic. This was statistically different than the sleep pattern of males (p = 0.019, Cramer V = 0.161) who tend to have more uniform and amalgamated patterns. No association was found between various age groups or occupations having inclination to a certain type of sleep pattern.

The people in our study tend to have duration of siesta e.g, 1–2 hours of sleep after waking at dawn and during afternoon (58% and 77.6% respectively). No gender-based discrepancy was observed in the duration of siesta. Furthermore, people who woke up for morning or midnight prayers tend to have more biphasic-dawn or polyphasic sleep patterns (p < 0.001, Cramer V = 0.398). Please refer to Table 2 for more information.

**Table 2:** Sleep Pattern in Relation to Gender, Age, and Employment.


CHARACTERISTICS	N	SLEEP PATTERNS					OR (95% CI)	P-VALUE

			MONOPHASIC NO (%)	BIPHASIC-SIESTA NO. (%)	BIPHASIC-DAWN NO. (%)	POLY-PHASIC NO. (%)		

**Gender**	Males	129	60 (46.5)	19 (14.7)	34 (26.4)	16 (12.4)	Siesta: 0.50 (0.27–0.94) Dawn: 1.21 (0.68–2.14) Poly: 0.52 (0.26–1.02)	0.031 0.524 0.055

	Females	255	95 (37.3)	61 (23.9)	49 (19.2)	50 (19.6)	Reference	–

**Age range**	18–34	325	131 (84.5)	69 (86.3)	71 (85.5)	54 (81.8)	Siesta: 0.83 (0.18–4.00) Dawn: 1.85 (0.40–8.56) Poly: 0.91 (0.19–4.36)	0.821 0.433 0.907

	35–49	43	17 (11.0)	8 (10.0)	9 (10.8)	9 (13.6)	Siesta: 0.97 (0.19–4.90) Dawn: 1.04 (0.21–5.30) Poly: 1.00 (0.20–5.00)	0.965 0.960 0.998

	50–64	16	7 (4.5)	3 (3.8)	3 (3.6)	3 (4.5)	Reference	–

**Employment**	Student	261	104 (67.1)	59 (73.8)	54 (65.1)	44 (66.7)	Siesta: 1.66 (0.35–7.81) Dawn: 0.31 (0.08–1.15) Poly: 0.79 (0.19–3.33)	0.521 0.079 0.744

	Employed	95	42 (27.1)	17 (21.3)	20 (24.1)	16 (24.2)	Siesta: 1.29 (0.31–5.35) Dawn: 0.33 (0.10–1.13) Poly: 0.79 (0.21–2.92)	0.722 0.077 0.721

	Unemployed	28	9 (5.8)	4 (5.0)	9 (10.8)	6 (9.1)	Reference	-


Most of our participants slept a total of 6–8 hours of sleep everyday (58.5%) followed by a small number of people who slept 8–10 hours (25.5%). There was no association between male v/s female (p = 0.640), age of the respondent (p = 0.641), or with the employment status (p = 0.716) with the number of daily sleep hours. Furthermore, no specific sleep pattern accounted for an increased or decreased duration of sleep compared to others (p = 0.527). Refer to Table 3 for further information.

**Table 3:** Duration of Sleep Characteristics Between the Four Types of Sleep Patterns.


DURATION OF SLEEP IN 24 HOURS	N	SLEEP PATTERNS				OR (95% CI)	p-VALUE

		MONOPHASIC NO (%)	BIPHASIC-SIESTA NO (%)	BIPHASIC-DAWN NO (%)	POLYPHASIC NO (%)		

Less than 6 hours	37	16 (10.3)	7 (8.8)	7 (8.4)	7 (10.6)	Siesta: 0.39 (0.11–1.43) Dawn: 0.88 (0.20–3.90) Poly: 0.88 (0.20–3.90)	0.121 0.861 0.861

6–8 hours	224	92 (59.4)	43 (53.8)	53 (63.9)	36 (54.5)	Siesta: 0.42 (0.15–1.15) Dawn: 1.15 (0.33–4.09) Poly: 0.78 (0.22–2.76)	0.091 0.824 0.721

8–10 hours	98	39 (25.2)	21 (26.3)	19 (22.9)	19 (28.8)	Siesta: 0.48 (0.16–1.42) Dawn: 0.97 (0.26–3.65) Poly: 0.97 (0.26–3.65)	0.185 0.969 0.969

More than 10 hours	25	8 (32.0)	9 (36.0)	4 (16.0)	4 (16.0)	Reference	–


The most common bedtime for participants was 12am-2am (47.1%) on weekdays, with only a small number of people waking up past 2 am (17.7%). However, it is during weekends, the number of people sleeping past 2 am almost doubled n = 142 (36.9%) where people tend to have more delayed bedtime compared to weekdays (p < 0.001, Cramer V = 0.572). A similar effect can also be observed in wake-up timings where most people woke up at 6am-8am on weekdays (51.3%) but the number decreased as the weekend arrived (p < 0.001, Cramer V = 0.451). Further information can be found on Table 4.

**Table 4:** Wake-up and Sleep Timings During Weekdays and Weekends.


TIMINGS	RANGES	WEEKDAYS	WEEKENDS	P-VALUE

**Wake-up timings**				

Before	4 AM	3 (0.8)	1 (0.3)	<0.001

Between	4 AM–6 AM	66 (17.2)	23 (6.0)	

	6 AM–8 AM	197 (51.3)	32 (8.3)	

	8 AM–10 AM	62 (16.1)	96 (25.0)	

	10 AM–12 AM	35 (9.1)	139 (36.2)	

	12 PM–2 PM	13 (3.4)	74 (19.3)	

After	2 PM	8 (2.1)	19 (4.9)	

**Sleep timings**				

Before	8 PM	1 (0.3)	2 (0.5)	<0.001

Between	8 PM–10 PM	15 (3.9)	10 (2.6)	

	10 PM– 12 PM	119 (31.0)	58 (15.1)	

	12 AM–2 AM	181 (47.1)	172 (44.8)	

	2 AM– 4 AM	52 (13.5)	100 (26.0)	

After	4 AM	16 (4.2)	42 (10.9)	


### Daytime Sleepiness & Sleep Patterns

Daytime Sleepiness was assessed using ESS scores. The mean score of samples was 7.8 ± 3.82. Furthermore, about a quarter of the population (23.69%) reported scores > 10 falling into the “mild” and “severe” daytime sleepiness categories.

Associations were drawn between sleep patterns and overall, ESS scores, using Kruskal-Wallis H test which showed biphasic-dawn sleepers had the least daytime sleepiness (7.18 ± 3.85) while the biphasic-siesta sleepers had the most (8.82 ± 3.744) daytime sleepiness (p = 0.024). The daytime sleepiness in monophasic sleepers compared to biphasic-dawn (p = 0.189) or polyphasic (p = 0.845) was non-significant.

Another key association showed men (6.89 ± 3.65) having an overall lower daytime sleepiness (p < 0.001) compared to the women (8.25 ± 3.829). And students reported the highest daytime sleepiness (8.4 ± 3.49) compared to people from other professions e.g., employed (6.56 ± 4.28) or unemployed (6.36 ± 3.851) (p < 0.001). Refer to Table 5.

**Table 5:** Daytime Sleepiness (ESS) and Sleep Quality (PSQI) Comparison for variables.


CHARACTERISTICS	N = 384	ESS MEAN (95% CI)	*p-VALUE*	PSQI MEAN (95% CI)	*p-VALUE*

**Sleep Patterns**			0.024		0.013
	
**Monophasic**	155	7.63 (7.06–8.20)		6.03 (5.55–6.51)	
	
**Biphasic (Siesta)**	80	8.82 (8.00–9.64)		6.94 (6.28–7.60)	
	
**Biphasic (Dawn)**	83	7.18 (6.35–8.01)		5.42 (4.82–6.02)	
	
**Polyphasic**	66	7.73 (6.74–8.72)		6.52 (5.72–7.32)	

**Gender**			0.001		0.000
	
**Male**	129	6.89 (6.26–7.52)		5.22 (4.75–5.69)	
	
**Female**	255	8.25 (7.78–8.72)		6.65 (6.26–7.04)	

**Employment Status**			0.000		0.096
	
**Student**	261	8.40 (7.98–8.82)		6.37 (6.02–6.72)	
	
**Employed**	95	6.56 (5.70–7.42)		5.86 (5.19–6.53)	
	
**Unemployed**	28	6.36 (4.93–7.79)		5.36 (4.06–6.66)	

**Waking up for Dawn Prayer**					0.018

**Yes**	211	–	–	5.87 (5.45–6.29)	

**No**	173	–	–	6.54 (6.09–6.99)	


### Sleep Quality & Sleep Patterns

The sleep quality of the participants was calculated using PSQI scores, where the mean score of our samples was 6.17 ± 3.08 (>5 score represents poor sleep quality).

As shown in Table 5, the PSQI scores of various sleep patterns were significantly different (p = 0.013) The biphasic-siesta sleepers reported the worst sleep quality (6.94 ± 3.01) while people having biphasic-dawn sleep pattern had the best (5.42 ± 2.8).

Sub-analysis was performed using Mann Whitney U test which showed no statistical difference between the overall sleep qualities between biphasic-dawn sleepers compared to monophasic (p = 0.115) or polyphasic (p = 0.06) or between monophasic sleepers against polyphasic (p = 0.456).

The average PSQI scores of men were significantly better compared to women (p = 0.0001). Men scored a mean of 5.22 ± 2.70 compared to females who scored 6.17 ± 3.08.

When participants were divided according to the students and employment status, the unemployed individuals in the current study had the best overall PSQI scores (5.36 ± 3.52) while the students had the worst (6.37 ± 2.91), but this association was non-significant (p = 0.096) (Table 5).

The association between the sleep quality among people who wake up for morning prayers against people who do not, was performed using Mann-Whitney U test. The results came out significant (p = 0.018). The people who woke up for morning prayers had a better PSQI scores (5.87 ± 3.11) compared to whose do did not (6.54 ± 3.00), with a possible weak correlation (spearman’s rho = +0.121) between the two variables (see Table 6).

**Table 6:** Waking up for Dawn Prayers.


WAKING UP FOR DAWN PRAYERS	N	SLEEP PATTERNS				p-VALUE

		MONOPHASIC NO (%)	BIPHASIC-SIESTA NO (%)	BIPHASIC-DAWN NO (%)	POLYPHASIC NO (%)	

**Yes**	211	45 (29.0)	21 (26.3)	82 (98.8)	63 (95.5)	<0.001

**No**	50	31 (20.0)	19 (23.8)	0 (0.00)	0 (0.00)	

**Sometimes**	117	77 (49.7)	36 (45.0)	1 (1.2)	3 (4.5)	

**Not applicable**	6	2 (1.3)	4 (5.0)	0 (0.0)	0 (0.0)	


## Discussion

The notable findings of this cross-sectional study showed that most of the young adults in Karachi, Pakistan region of South-Asia has monophasic sleep patterns, with almost half of the study size following this routine. Segmented sleep patterns, and circadian irregularities might be attributed to an increased serum cortisol levels, poor metabolism, and an overall increased risk of cardiovascular diseases [14]. Therefore, monophasic sleep pattern may have a protective effect e.g., lower incidence of said disorders.

Women were more likely to have segmented sleep patterns and take afternoon naps than men, who tend to have more uniform sleep patterns. This may partly reflect the higher proportion of stay-home housewives in our sample, whose household obligations contribute to segmented sleep. Conversely, we found no difference in sleep patterns across various professions or age groups from our study. But the association may be inconclusive given the nature of student-majority data in our sample.

Our study showed that most of the population was getting the recommended amount of sleep every night (6–8 hours). National Sleep Foundation guidelines recommends that an average adult requires 7–9 hours of sleep per night, although individual differences may occur [15]. Furthermore, sleep duration remained constant across sleep patterns and between men and women.

Our sample population showed an increased duration of sleep compared to other international studies. A multi-national survey on 35,327 adults in ten countries showed certain regions do report higher durations of study – where the global average was around 7 hours and 34 minutes per 24 hours and some countries like the Portuguese, who slept a plentiful 8 hours and 24 minutes of sleep everyday [19]. A survey conducted on American population found an average American slept 6.5 hours – a number that is significantly lower compared to last four decades [16]. Japan reported to have an average of 6 hours and 53 minutes of sleep every night [17]. Gulf countries such as Oman showed majority of adults slept less than 7 hours every day [18].

Surprisingly, the monophasic sleep pattern is comparatively uncommon. Multiple studies have shown daytime nap or siesta is the most common pattern throughout the world – in the regions of Mediterranean, southern Europe, Spain, Southern Italy, many Hispanic American countries, the Philippines, parts of Africa, China, and Japan [18,20,21,22]. Daytime napping is also common in other communities e.g., Brazilian Native Terena adults, in Japan, and even in Muslic-centric Omani adults, afternoon siestas were the most common type of sleep pattern [18,20,23].

In terms of the quality of the sleep, our participants was above average – with about a quarter of people reporting to have ESS scores > 10 and a mean score of 6.5 on PSQI. Furthermore, we found significant difference in the sleep quality and associated patterns. People who usually napped in the afternoon (biphasic-siesta pattern) reported to have the worst sleep quality and the highest daytime sleepiness on PSQI and ESS scales respectively. Compared with other sleep patterns, people who woke up for morning prayers reported to have the best sleep quality and the least daytime sleepiness overall. People with monophasic and polyphasic patterns fell between the spectrum, with the difference in their scores being non-significant (only biphasic-siesta scores were significantly different than the other sleep patterns. Our findings are in association with an Omani study which showed biphasic-siesta sleep pattern had to worst quality and the polyphasic pattern had the highest rates of daytime sleepiness [18]. Furthermore, the population that reported ESS > 10 was higher as well – about one-third compared to our study’s one-fourth, possibly due to segmented sleep patterns being more common in the former study.

A similar case can be made for another finding in our study which showed males tend to have better sleep quality and less daytime sleepiness than females – where the latter tend to have more segmented sleep patterns than the former. The sleep quality in Pakistani adults was significantly better compared to other regions as well. A study conducted in America showed that about 25% of the population reported excessive daytime sleepiness [24] or a South Korean study which found 12.2% of the population having excessive daytime sleepiness [25].

One very fascinating finding from our study was how the people waking up for morning prayers (and sleeping afterwards) tend to have the best sleep quality and the least prevalence of daytime sleepiness. Furthermore, there was a weak positive correlation in waking up in the morning and having a better sleep quality. Such a finding has not been reported previously in literature –on the contrary, studies like Bahammam et al reported no difference in sleep architecture or daytime sleepiness in the consolidated (monophasic) and split-sleep schedules (biphasic-dawn) when the total sleep duration was maintained [10]. However, Bahammam’s study was a case-control, simulated under observational scenario while we conducted the study strictly through an individualized questionnaire form. Moreover, the mean age group in that study was far older than ours, leading us to believe people who woke up in the morning did so out of their body’s natural circadian rhythm instead of a laboratory-simulated condition. Further studies are needed to support or negate our claim of waking up in the morning might have a positive impact in the overall sleep routine.

Notwithstanding the findings from our study, our results may be subjected to recall bias, as most of the data gathered was through a questionnaire. Furthermore, participants undergoing one-on-one interviews might have been subjected to Hawthorne effect. Both were tackled by keeping the questionnaire subjected to their most recent sleep schedules, with standardized, and previously tested methods (ESS & PSQI scales) of data collection, while also keeping out database online, which prevented data entry errors. One way our data could have been more precise is if had taken age and sleeping hours as continuous variables instead of nominal. However, similar effects could be observed in both the conditions, so it did not hinder our final analysis. Lastly, one major drawback of our study was a small population size and diversity due to lack of manpower and resources to conduct a large-scale study. The high proportion of students affects the generlisability of results. Studies with robust sampling techniques might be able to draw clearer and more valid associations between sleep patterns, quality, and age-gender and lifestyle relationships in local population. Studies should follow advocacy to educate young adults on the importance of adequate sleep and integrating sleep hygiene awareness into university health programs and inclusion of sleep screening in routine student and workplace check-up. Alarming levels of the frequency of less-than-optimal sleep signal an emerging public health concern related to stress, screen overuse and lifestyle imbalance among population which need to be further assessed. Further studies identifying avoidable risk factors related to improper sleep among local population are advised.

This study simultaneously examines sleep patterns and quality in urban population of Pakistan’s biggest city where awareness about sleep quality were found below par. We hope more studies are conducted in this subject so government and medical institutions, especially in the said region, can have tailored approach and policies to optimize the functioning of their people.

## Conclusion

The Urban young adult population in the port city of Karachi in Pakistan tends to have a single-phase (monophasic) sleep pattern most commonly. The people sleeping after waking up early morning prayers (biphasic-dawn) were most likely to have the best sleep quality and least daytime sleepiness. Women tend to sleep in more segmented sleep than men, while men had better sleep quality overall.

## Disclosures

The permission to perform data collection was obtained from Institutional Review Board (IRB) – Dow University of Health Sciences [Ref ID: IRB-3002/DUHS/Approval/2023/312].
